# Is the Measurement of Accessory Pathway Refractory Period Reproducible?

**DOI:** 10.1016/s0972-6292(16)30501-0

**Published:** 2012-05-20

**Authors:** Celine Oliver, Beatrice Brembilla-Perrot

**Affiliations:** Department of cardiology, University Hospital of Brabois, Vandoeuvre, France

**Keywords:** Wolff-Parkinson-White syndrome, accessory pathway, electrophysiology

## Abstract

**Introduction:**

Short accessory pathway (AP) effective refractory period (ERP) is one of the risk factors in Wolff-Parkinson-White syndrome (WPW). The purpose of study was to evaluate the reproducibility of APERP measurement during a same electrophysiological study (EPS).

**Methods:**

EPS consisted of 2 APERP measurements performed prospectively in 77 patients for a WPW in control state (CS) at a cycle length of 400 ms (n=76) and after isoproterenol (n=56).

**Results:**

In CS, 18 patients (24 %) had the same APERP at both measurements; 41 (54.6 %) had differences from 10 to 40 ms, 17 (22.4 %) had differences > 40 ms. Among 45 patients with initial APERP > 240 ms, 7 had an APERP ≤ 240 ms at 2nd study. Among 31 patients with initial APERP ≤ 240 ms, 5 had an APERP > 240 ms at 2nd study. Pearson's product-moment correlation was 0.75. After isoproterenol, 5 patients (9 %) had the same APERPs; 37 (66 %) had differences from 10 to 40 ms and 14 had differences > 40 ms. Among 38 patients with initial APERP > 200 ms, 12 had an AP ERP ≤ 200 ms at 2nd study. Among 18 patients with initial APERP ≤ 200 ms, 10 had still APERP ≤ 200 ms at 2nd study. Pearson's product-moment correlation was 0.54.

**Conclusion:**

There are important variations of APERPs during EPS mainly after isoproterenol infusion. Therefore the values of APERPs should be interpreted cautiously.

## Introduction

The prevalence of a typical Wolff-Parkinson-White (WPW) pattern is estimated to be 0.1% [1]. Sudden cardiac death might be the first clinical manifestation of the WPW syndrome in previously asymptomatic individuals [2]. A recent study [3] reports a sudden cardiac death risk of 0.02%/patient/year. But the risk of sudden death is increased in some clinical situations. So it is important to detect high risk form for developing fatal arrhythmic events, in order to propose a curative treatment by radiofrequency ablation in a selected asymptomatic population [4]. The WPW syndrome is considered as representing a risk of life-threatening arrhythmic events when the following association is observed [5]: 1. Sustained atrial fibrillation is induced [6] ; 2. The shortest RR interval between preexcited beats is less than or equal to 240 ms n the control state in adults [6] (or an anterograde effective refractory period of accessory pathway (APERP) is less than or equal to 240 ms) [7,8] Or the shortest RR interval between preexcited beats is less than 200 ms or an AP ERP is less than 220 ms (or 200 ms depending on authors) after isoproterenol infusion [9,10]. 

Other factors are discussed such as the presence of multiple accessory pathways and the male gender. Therefore, the measurement of the AP ERP during an electrophysiological study is important. Actually AP ablation is largely indicated and a short APERP might be sufficient to indicate AP ablation. The aim of this study is to evaluate the reproducibility of the measurement of AP ERP during the same electrophysiological study.

## Methods

### Population

Seventy seven patients, 56 males (73 %) and 21 females (27 %) aged from 8 to 57 years (mean age 31 ± 12 years), with a ventricular preexcitation syndrome were consecutively included in this study; 46 patients were asymptomatic (60 %), 20 patients had palpitations (27 %), 7 patients had history of unexplained syncope (7 %), 2 had spontaneous documented atrial fibrillation (3 %). Only 2 patients (3 %) had a history of life-threatening arrhythmic events (ventricular fibrillation or atrial fibrillation with fast ventricular conduction).

Accessory pathway was septal located in 51 patients (left 23, right 28). Three patients had an anteroseptal location (4 %); 19 patients had a left lateral accessory pathway location (25 %) and 4 a right lateral location (5 %).

### Methods

This was a prospective study performed between 2004 and 2010 after informed consent. An electrophysiological study was performed in patients not sedated and after cessation of treatment. Patients were studied by transesophageal route and/or intracardiac route [11]. The classical protocol was as follows: 1) Incremental atrial pacing was performed until the highest rate conducted 1/1 through the accessory pathway and/or the AV node. 2) Programmed atrial stimulation in control state at basic cycle lengths of 400 ms was performed: one atrial extrastimulus was delivered after 7 paced atrial stimuli at a cycle length of 400 ms from 390 ms until the AP refractory pathway or the atrial effective refractory period with decrement of 10 msec. 

The disappearance of the pattern of preexcitation indicated when the APERP was reached. The longest A1A2 that fails to conduct at the atria was considered as the effective APERP. This protocol was reproduced again after several minutes to study the reproducibility of the measure of anterograde effective refractory period.

The method was used to induce supraventricular tachycardia. The protocol was performed during 80 consecutive electrophysiological studies. Three patients were excluded from this protocol because sustained atrial fibrillation was induced at first programmed atrial stimulation and flecainide injection was required to stop it. In one patient the AP did not conduct in control state and the measurement of APERP was made only after isoproterenol.

In the absence of induction of a tachycardia conducted through the accessory pathway at a rate higher than 250 bpm, isoproterenol (0.02 to 10 μg.min-1) was then infused to increase the sinus rate to at least 130 bpm and the pacing protocol was repeated. APERP was determined twice at a basic cycle length of 400 ms. Isoproterenol was infused in only 56 patients: 9 patients did not require isoproterenol infusion because they had an electrophysiological malignant form in control state with the induction of an atrial fibrillation and short cycle lengths conducted by AP. Two other patients remained in atrial fibrillation without signs of malignancy after second programmed stimulation in control state. Poorly-tolerated orthodromic tachycardia was induced in 5 symptomatic patients during the basal study and catheter ablation was indicated without evaluation after isoproterenol. In 6 children, isoproterenol infusion was poorly-tolerated and programmed atrial stimulation was not repeated.

### Definitions

Conduction over the accessory atrioventricular connection was evaluated by the measurement of the maximal heart rate with a 1 to 1 conduction over the accessory connection and the shortest atrial tachycardia cycle length at which there was 1 to 1 conduction over the accessory connection. A short APERP was defined in the present study as less than or equal to 240 ms in control state (CS) and less than or equal to 200 ms after isoproterenol.

### Statistical analysis

Results were presented as mean and standard deviation and compared with the paired Student t test. A value of p < 0.05 was considered to be significant. Correlations were performed between 2 determinations of AP ERP and agreement was expressed according to the Pearson's correlation coefficient. It was obtained by dividing the covariance of the two variables by the product of their standard deviations.

## Results

### Reproducibility of APERP determination in control state at basic cycle lengths of 400 ms (Figure 1):

**Figure 2:**
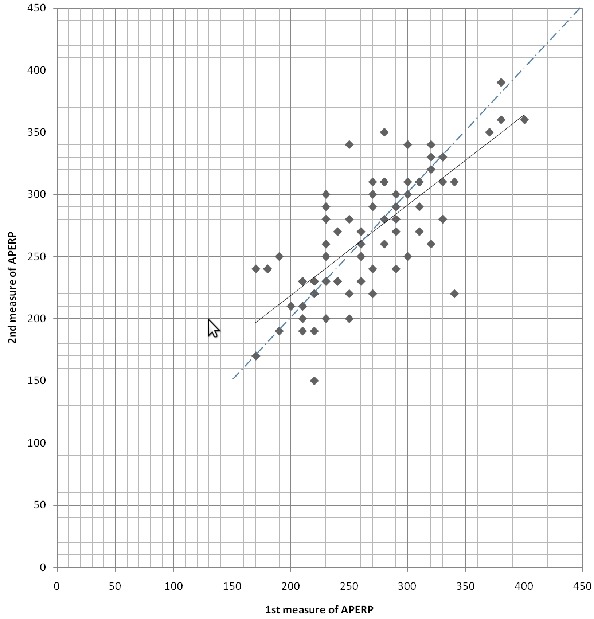
Representation of the dispersion of the 2 different measurements of accessory pathway refractory periods with an atrial pacing at basic cycle lengths of 400 ms

The mean fastest cardiac rate conducted by the accessory pathway was 214±55 bpm in control state and the ranges were 100 and 300 bpm. The mean sinus cycle length did no differ significantly during both measurements (805±12 vs 819±11msec). The dispersion of the two measurements of APERP is represented on a graphic in Figure 2. The mean value of APERP at the first study was 264±52 ms. Mean value of the second measure was 265±50 ms (NS). However, there were important individual changes of APERP values between both studies.

At the first determination, APERP was more than 240 ms (mean 299± 37) in 45 patients (59 %); mean value was the same in the second study (299± 37 ms); APERP became less than or equal to 240 ms at second measurement in 7 patients (15.5 %) and remained more than 240 ms in 38 patients (84.5 %). At the first APERP determination, 31 patients (41 %) had an APERP less than or equal to 240 ms: 24 patients had an APERP less than or equal to 240 ms at both studies (77 %); 7 patients had an APERP more than 240 ms at second study (22.5 %). The mean absolute difference between the two trials was 25.7 ms (±25.2 ms). The variations are reported in Table 1.

Twenty-eight measurements (37%) were shorter in the second trial than in the first with a mean difference of 34±24 ms. Thirty measurements (40 %) were shorter in the first trial than in the second, with a mean difference of 34±23 ms. The Pearson's product - moment correlation was 0.75. 

### Reproducibility of APERP determination after infusion of isoproterenol (Figure 1):

**Figure 3:**
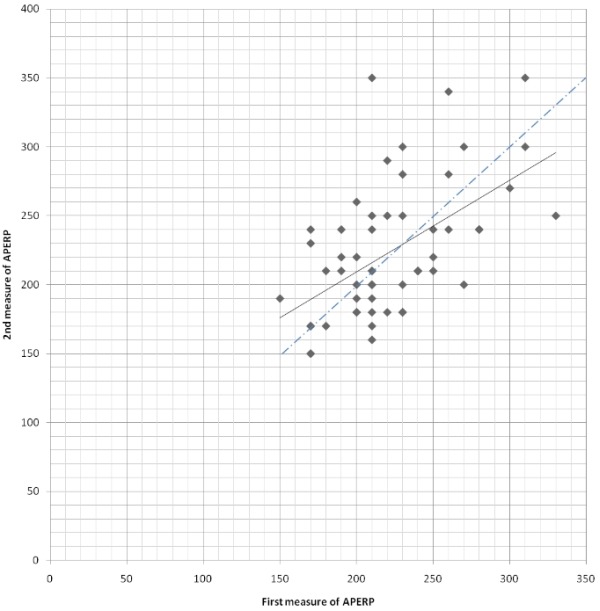
Representation of the dispersion of the 2 different measurements of accessory pathway refractory periods after isoproterenol infusion

The mean fastest cardiac rate conducted by the accessory pathway was 265±18 bpm after isoproterenol infusion. The mean cycle length between 2 measurements does not differ significantly (475±12 mesc vs 479±14 ms). The dispersion of the two measurements with isoproterenol infusion was represented on a graphic in Figure 3.

The mean value of the first measurement was 223±40 ms. Mean value of the second measurement was 224±48 ms (NS). Individual data differed. At the first APERP determination, 18 APERPs (32%) were less than or equal to 200 ms; 10 measures (56%) were less than or equal to 200 ms and reproducible at 2 measurements; 8 APERPs were more than 200 ms at the second determination (46%). The mean absolute difference between two trials was 34±25.4 ms. Among 38 patients with initial APERP more than 200 ms, 12 measures were less than or equal to 200 ms at the second determination (33%). The individual variations are reported in Table 1.

For 30 patients (53.4 %) the APERP was shorter at the second measurement than at the first, with a mean difference of 30±18 ms. For 21 patients (37.5 %) the first measure of APERP was the shortest with a mean difference of 47.6 ± 28.4ms. The Pearson's product - moment correlation was 0.54.

### Reproducibility according to the route (intracardiac or transoesophageal) of electrophysiological study:

Fourteen patients underwent transesophageal study and later intracardiac study to perform the accessory pathway ablation. The initial mean values of APERP measured at esophageal study were significantly shorter than the values measured at intracardiac study, but other measurements did not differ significantly. The mean variations were similar either in control state (31±29 ms and 30±27 ms) or after isoproterenol (26±16 and 24±15 ms) (Table 2).

### Reproducibility according to the age:

The mean age of our population was 31±12 years. In the youngest population (30 years old or less), representing 36 patients, the Pearson correlation coefficient was 0.675 in control state and 0.58 after isoproterenol infusion. In the oldest population (more than 30 years old), representing 36 patients, the Pearson correlation coefficient was 0.78 in control state and 0.46 after isoproterenol infusion. The subgroups according to the age of the patient were too small to verify possible relationships between the age and the variability of APERP.

### Reproducibility according to the location of accessory pathway:

There were no significant differences of APERP according to the location of AP: AP was left-sided in 19 patients and the variations were 27±25 ms in control state and 25±13.5 ms after isoproterenol. AP was septal in 54 patients and the variations were 24±25.5 ms in control state and 35±27 ms after isoproterenol. AP was right lateral side in only 4 patients and variations (42.5±22 ms in control state and 35±21 ms after isoproterenol) can not be interpreted.

Among the 2 patients who have presented life-threatening arrhythmic events and 2 other patients with syncope and malignant form of preexcitation syndrome at electrophysiological study; all had a maximal heart rate with a 1 to 1 conduction over the accessory pathway was more than 240 bpm in control state during induced sustained atrial fibrillation. AF stopped spontaneously and the protocol was performed in these patients. Two patients had an APERP of 250 and 260 ms at the first measurement and a short value (210 and 230 ms) at the second measurement. Other patients had a short value (190 ms) at both measurements.

## Discussion

We reported important variations of APERPs during electrophysiological study mainly after isoproterenol infusion. This may mean that ERP measurement with isoproterenol cannot be relied upon for assessing risk. The variations could be partially explained by the various conditions of the autonomic nervous system. The vagal and sympathetic activities constantly interact, and modulate the electrophysiological behaviour of the heart under different conditions such as postural changes, effort, and psychological activation [12,13]. A case of emergence of bidirectional accessory pathway conduction in adulthood reported by Nabar A [14] highlights the possible emergence of an AP in adult life.

Fenici and al [15] studied the reproducibility of transesophageal pacing in patients with Wolff-Parkinson-White syndrome by two transesophageal electrophysiological studies which were performed approximately 3 months apart. The results of this study demonstrated that changes of autonomic modulation could induce significant variations in the electrophysiological parameters that were commonly used to define arrhythmogenic risk in WPW patients. But the intraindividual coefficient variation was less than 10% in all patients [16]. It was clear that APERP, which is dependent on the autonomic tone, could vary during electrophysiological study, depending on the initial stress of the patient, the occurrence of a vagal reaction or the induction of a tachycardia.

As showed by Castellanos et al [16], if the accessory pathway has a short effective refractory period, very high ventricular rates can occur after the onset of atrial fibrillation. Under these circumstances, atrial fibrillation can be a life-threatening arrhythmia. The incidence of atrial fibrillation in the Wolff-Parkinson-White syndrome is approximately 11.5% [17]. Wellens had compared the length of the refractory period of the accessory pathway with the ventricular frequency during spontaneous or electrically induced atrial fibrillation [10] and reported a good correlation between the 2 values.

The classical values of 240 ms in controls state and 200 ms after isoproterenol as cut-off for the evaluation of the arrhythmic risk should be discussed and the values of APERP's interpreted carefully in association with other data of electrophysiological study. Male gender, young age, sport, septal accessory pathway (AP), multiple accessory pathways, short AP refractory period, atrial fibrillation (AF) were reported as risk factors of sudden death in WPW syndrome [6,18,19]. Pappone and al considered only the induction of a re-entrant tachycardia or atrial fibrillation as a risk factor of arrhythmic events in asymptomatic patients. The same group reported recently 3 risk factors of events and sudden death, the inducibility of tachycardia, a short AP refractory period (less than 250 ms in adults, 240 ms in children) and the presence of multiple pathways [7,8].

## Limitations of the study

Some of our studies were performed by transesophageal route. However 14 of these patients had later an intracardiac study and the variations of APERPs were similar. Nanthakumar et al [20] have compared transesophageal route and intracardiac rout at 24 hour interval. Despite adequate reproducibility, transesophageal atrial stimulation was shown to fail to predict the AP-ERP by intracrdiac stimulation. Differences in stimulus latency was responsible, in part, for the disagreement.

Three patients were excluded after induction of atrial fibrillation at the first measurement, requiring flecainide to stop it. We can not prove the role of autonomic nervous system because no changes in sinus cycle length were found in the present study. We have not studied the reproducibility of the ventricular rate during induced atrial fibrillation, because AF was not always inducible or required flecainide to stop it.

Only 3 patients had a spontaneous malignant form. More, the ages of our population are heterogeneous and a difference between younger and older patients can not be excluded. The data are only applicable to non sedated patients. Accessory pathways were mainly septal located probably because the majority of our patients were asymptomatic. Similar data were noted by our group [11] in a larger cohort of 645 patients with a preexcitation syndrome. At least, our statistical analysis can be debatable.

## Conclusion

In conclusion this prospective study reports important variations of APERPs during electrophysiological study mainly after isoproterenol infusion. The study highlights the limitation of measurement of ERP as a predictor of the future risk. The APERP in patients with WPW should be interpreted with carefulness in association with other data of electrophysiological study. We suggest repeating the measurement in patients complaining of palpitations or syncope to avoid missing short values of APERP.

## Figures and Tables

**Figure 1:**
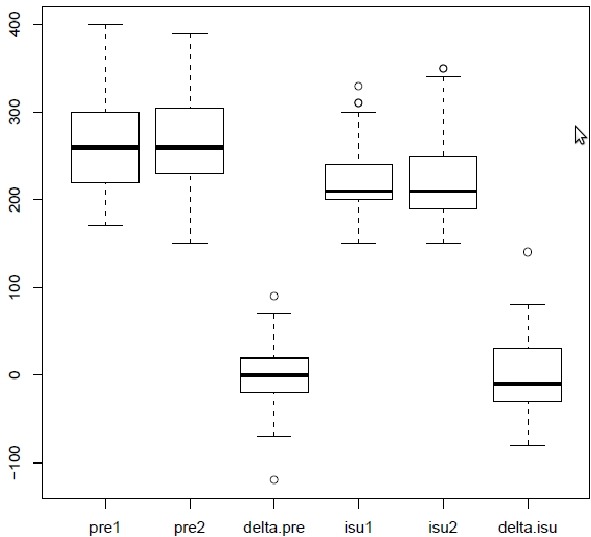
Representation with box plot of the value's variability around the median. PRE= AP effective refractory period (APERP); isu: isooproterenol

**Table 1:**
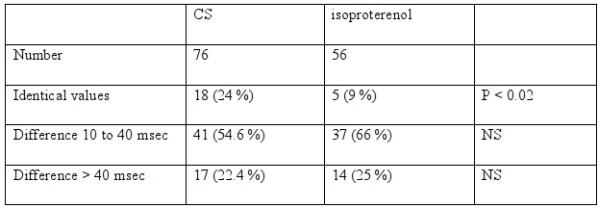
Mean values of variations of APERP in control state (CS) or with isoproterenol

**Table 2:**
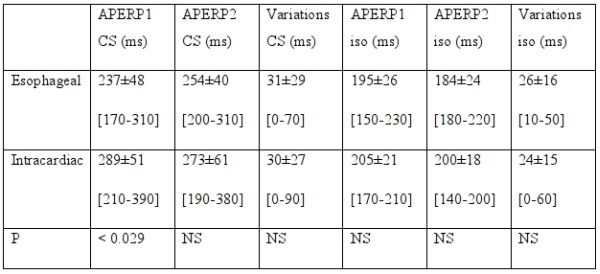
Data obtained in patients studied by esophageal route and then by intracardiac route

APERP1: AP effective refractory period at first measurement; APERP2: APERP at second measurement CS: control state; iso: isoproterenol. Data are expressed as mean ± standard deviation and ranges
